# Display of Multimeric Antimicrobial Peptides on the *Escherichia coli* Cell Surface and Its Application as Whole-Cell Antibiotics

**DOI:** 10.1371/journal.pone.0058997

**Published:** 2013-03-14

**Authors:** Ju Ri Shin, Ki Jung Lim, Da Jung Kim, Ju Hyun Cho, Sun Chang Kim

**Affiliations:** 1 Department of Biological Sciences, Korea Advanced Institute of Science and Technology, Daejeon, Republic of Korea; 2 Department of Biology, Research Institute of Life Science, Gyeongsang National University, Jinju, Republic of Korea; Belgian Nuclear Research Centre SCK/CEN, Belgium

## Abstract

Concerns over the increasing emergence of antibiotic-resistant pathogenic microorganisms due to the overuse of antibiotics and the lack of effective antibiotics for livestock have prompted efforts to develop alternatives to conventional antibiotics. Antimicrobial peptides (AMPs) with a broad-spectrum activity and rapid killing, along with little opportunity for the development of resistance, represent one of the promising novel alternatives. Their high production cost and cytotoxicity, however, limit the use of AMPs as effective antibiotic agents to livestock. To overcome these problems, we developed potent antimicrobial *Escherichia coli* displaying multimeric AMPs on the cell surface so that the AMP multimers can be converted into active AMP monomers by the pepsin in the stomach of livestock. Buf IIIb, a strong AMP without cytotoxicity, was expressed on the surface of *E. coli* as Lpp-OmpA-fused tandem multimers with a pepsin substrate residue, leucine, at the C-terminus of each monomer. The AMP multimers were successfully converted into active AMPs upon pepsin cleavage, and the liberated Buf IIIb-L monomers inhibited the growth of two major oral infectious pathogens of livestock, *Salmonella enteritidis* and *Listeria monocytogenes*. Live antimicrobial microorganisms developed in this study may represent the most effective means of providing potent AMPs to livestock, and have a great impact on controlling over pathogenic microorganisms in the livestock production.

## Introduction

Modern livestock production systems have continually used antibiotics and antimicrobial compounds to either prevent and treat infectious diseases or improve weight gain and feed utilization in animals. In fact, in-feed administration of non-therapeutic doses of antibiotics was found to increase the performance of growing livestock [1]. However, the indiscriminate non-therapeutic use of antibiotics in the livestock production has a negative impact not only on livestock but also on public health and food safety, as it promotes the rapid development of multidrug-resistant bacteria that do not respond to current antibiotics, thus further endangering human lives [2]–[5]. These antibiotic-resistant bacterial strains and associated genes have the potential to impart their resistance traits to disease-causing bacteria. Humans acquire these resistant bacteria either through direct contact with infected livestock or contaminated food or water. Therefore, many countries have banned the administration of conventional antibiotics (at non-therapeutic doses), as feed additives to livestock [6], [7]. The rapid increase in the number of antibiotic-resistant pathogenic microorganisms due to antibiotic overuse as well as the limited number and low availability of effective antibiotics for livestock has led to numerous researchers focus on the development of alternatives to conventional antibiotics [8]–[10].

Antimicrobial peptides (AMPs) have been considered as one of the most promising alternative antibiotics because of their strong antimicrobial activity and microorganismal eradication with a little opportunity of developing resistance. AMPs are produced by all classes of life, and play key roles in primary host defense against infection by pathogenic microorganisms. While commonly prescribed antibiotics operate on specific intracellular targets, AMPs physically compromise bacterial membrane integrity by disrupting essential components within the cells, thereby causing bacteria difficult to develop resistance [11], [12]. Despite their potential as alternative antibiotics, the use of AMPs in the livestock has a limitation due to their cytotoxicity and high production cost. Owing to their membrane lytic mechanism, several AMPs represent toxicity towards eukaryotic cells at higher concentrations [11]. Moreover, chemical synthesis of AMPs on a large scale and in a pure enough form to be used for livestock is extremely expensive. The production of recombinant AMPs is also expensive because the host cell needs to be protected against the potent action of the peptides. Thus, the livestock industry has not shown a great deal of interest in developing antibiotic alternatives based on AMPs.

Buf IIIb (RVVRQWPIGRVVRRVVRRVVR), a potent cell-penetrating AMP which does not cause damage to mammalian host cells up to 200 µg/ml, is a promising candidate for alternative antibiotics [13]. In this study, we developed the most cost effective means of providing AMPs to livestock by consisting rapid-acting and potent antimicrobial *Escherichia coli* displaying multimeric Buf IIIb on the cell surface. Buf IIIb was expressed as Lpp-OmpA-fused tandem multimers on the surface of *E. coli*, with a pepsin substrate residue at the C-termini of each monomer [14]–[16]. Upon cleavage by pepsin, the Lpp-OmpA-fused tandem multimers displayed on the surface of *E. coli* were converted into active AMP monomers, and the liberated Buf IIIb-L monomers inhibited the growth of selective major oral infectious pathogens of livestock.

## Materials and Methods

### Bacterial strains, plasmids, and enzymes

*E. coli* XL1-Blue (Stratagene, La Jolla, CA, USA) was used as a host for sub cloning, and *E. coli* BL21 (DE3) (Invitrogen, Carlsbad, CA, USA) was used for gene expression. *E. coli* cells were grown in Luria-Bertani (LB) medium at 37°C, and ampicillin (50 µg/ml) was added for the growth of plasmid-containing cells. The pGEM-T easy vector (Promega, Madison, WI, USA) was used for sub cloning and multimerization of the Buf IIIb-L gene, and pET21c (Novagen, Madison, WI, USA) for the expression of the Lpp-OmpA-multimeric Buf IIIb-L fusion (LO-B_n_) proteins. Restriction enzymes were purchased from New England Biolabs (Beverly, MA, USA). Taq polymerase and porcine pepsin were purchased from Takara (Otsu, Japan) and Sigma (St. Louis, MO, USA), respectively. All enzymes were used according to the recommendations of suppliers. All recombinant DNA techniques were performed as described by Sambrook and Russell [17].

### Peptide synthesis

Peptides used in this work (Table 1) were chemically synthesized on a Milligen 9050 peptide synthesizer (Anygen, Kwangju, Korea). Synthesized peptides were purified to over 88% by reversed-phase high-pressure liquid chromatography (HPLC) on a Delta-Pak C18 column (3.9 mm×300 mm, Waters, Milford, MA, USA). The peptide content of lyophilized samples was determined by quantitative amino acid analysis with a Pico-tag system on a Beckman 121 MB amino acid analyzer (Beckman Coulter, Fullerton, CA, USA).

**Table 1:** Amino acid sequences of Buf IIIb derivatives.

Peptides	Amino acid sequences
Buf IIIb	RVVRQWPIGRVVRRVVRRVVR
Buf IIIb-L	RVVRQWPIGRVVRRVVRRVVRL
Buf IIIb-F	RVVRQWPIGRVVRRVVRRVVRF
Buf IIIb-Y	RVVRQWPIGRVVRRVVRRVVRY

### Antimicrobial activity

The antimicrobial activity of each peptide was determined against eight representative microorganisms, including Gram-positive and Gram-negative bacteria and fungi, using the broth micro dilution assay as described by Park *et al*. with a slight modification [18]. Briefly, mid-logarithmic phase cells were diluted to 1×10^5^ cfu/ml in 10 mM sodium phosphate buffer (NAPB), pH 7.4. Each well of 96-well propylene micro titer plates (Costar, Cambridge, MA, USA) was filled with 90 µl of the diluted cell suspension and 10 µl of serially diluted peptide samples. After incubation for 3 h, fresh medium (trypticase soy broth for bacteria and Saboraud' medium for fungi) was added to the mixture and incubated at 37°C (bacteria) or 30°C (fungi) for an additional 12 h. Inhibition of growth was determined by measuring the absorbance at 620 nm with a Model 550 Microplate Reader (Bio-Rad, Hercules, CA, USA). The lowest concentration of peptide that completely inhibited growth was defined as the ‘minimal inhibitory concentration’ (MIC). The MIC values were calculated as an average of two independent experiments performed in triplicate.

### Hemolysis and *in vitro* cytotoxicity assays

Hemolytic activity was assayed as described by Jang *et al*. [13]. The percentage of hemolysis was calculated using the following equation: Hemolysis (%) = (A_s_−A_0_)/(A_100_−A_0_)×100, where A_s_ is the absorbance at 567 nm of the sample, A_100_ is the absorbance of completely lysed human red blood cells (RBCs) in 0.2% Triton X-100, and A_0_ is the absorbance of zero hemolysis.

To analyze *in vitro* cytotoxic activity, HaCaT keratinocytes were cultured in 96-well plates (10^5^ cells/well) in Dulbecco's modified eagle medium (DMEM) with 10% FBS. After 24 h of incubation, cells were treated with each peptide (0–400 µg/ml) and incubated for another 24 h. Cells viability was measured with the 3-(4,5-dimethylthiazol-2-yl)-2,5-diphenyl tetrazolium bromide (MTT) assay using the CellTiter 96^®^ Non-radioactive Cell Proliferation assay Kit (Promega, Madison, WI, USA) according to the manufacturer' instructions. The percentage of cell viability was determined using the following equation: Viability (%) = (A_s_−A_0_)/(A_c_–A_0_)×100, where A_s_ is the absorbance of at 570 nm of the sample, A_c_ is the absorbance of control (no peptide addition), and A_0_ is the background absorbance. Each experiment was performed in triplicate, and repeated at least three times independently.

### Construction of expression vectors containing Lpp-OmpA-multimeric Buf IIIb-L fusion genes

**Figure 1:**
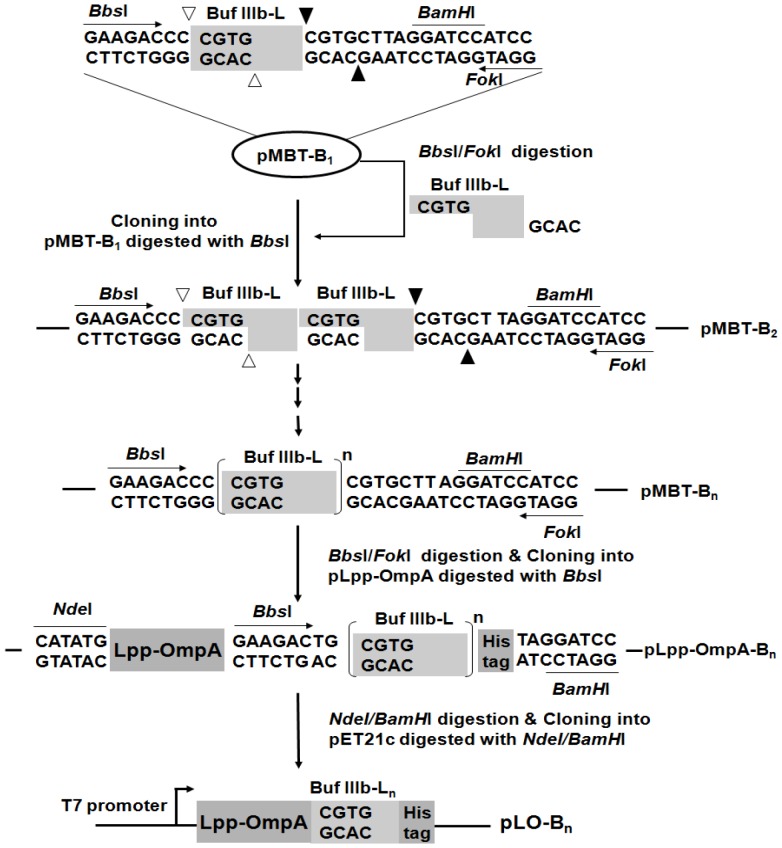
Schematic representation of the construction of Lpp-OmpA-multimeric Buf IIIb-L fusion genes. A monomeric Buf IIIb-L gene was dimerized by; (1) excision of the monomeric Buf IIIb-L insert by digestion with *Bbs*I and *Fok*I, (2) isolation of the fragment, (3) cloning into the original pMBT-B_1_ vector digested with *Bbs*I, generating pMBT-B2. These steps were repeated for the construction of tandem multimers of the Buf IIIb-L gene, generating pMBT-B_n_ (n = number of Buf IIIb-L genes). The *Bbs*I and *Fok*I fragments of pMBT-B_n_ were cloned into pLpp-OmpA digested with *Bbs*I, generating pLpp-OmpA-B_n_. The *Nde*I and *BamH*I fragments of pLpp-OmpA-B_n_ were then ligated into the expression vector pET21c digested with the same enzymes, generating pLO-B_n_ (n = 0, 1, 2, and 3). The *arrows*, *open triangles* and *closed triangles* indicate *Bbs*I cleavage sites and *Fok*I cleavage sites, respectively.

The overall scheme for the construction of the expression vectors is illustrated in Fig. 1. The gene encoding the Buf IIIb-L was synthesized using the following deoxyoligonucleotides (oligos): 5′-**GAAGAC**CCCGTGTTGTTCGTCAGTGGCCGATTGGTCGTGTCGTTCGCC GTGTTGTTCG-3′ and 5′-GGAT*G* *GATCC*TAAGCACGCAGACGAACGACGCGACGAA CAACACGGCGAACGACACGAC-3′ (restriction sites *Bbs*I, *Fok*I, and *BamH*I are indicated in the oligos as bold, underlined, and italic, respectively). The two oligos were annealed by PCR and ligated into the linear vector with 3′-T overhangs (pGEM-T vector) to generate pMBT-B_1_. The DNA fragment encoding the Buf IIIb-L monomer was isolated from pMBT-B_1_ after digestion with *Bbs*I and *Fok*I, and cloned into *Bbs*I-digested pMBT-B_1_, generating pMBT-B_2_ containing a Buf IIIb-L dimer. These steps were repeated for the construction of tandem multimers of the Buf IIIb-L gene, generating pMBT-B_n_ (n = number of Buf IIIb-L monomers).

To construct Lpp-OmpA-multimeric Buf IIIb-L fusion genes, an anchor protein encoding the chimeric Lpp-OmpA gene consisting of the signal sequence and the first nine amino acids of lipoprotein (Lpp), residues 46-159 of outer membrane protein OmpA, and His tag (histidine 6-mer), was amplified from *E. coli* chromosomal DNA using recombinant PCR with the two primer pairs, 5′-CGC**CATATG**AAAGCTACTAAACTGGTACTGGGCAACA ACAATGGCCCGACCCATGAAAAC-3′ (*Nde*I site indicated as bold)/5′-GCAAACACCGG AGAAACGCCGGTG-3′ and 5′-TTCTCCGGTGTTTGCTGGCGGTGTTG-3′/5′-CG**GGAT CC**TAGTGATGGTGATGGTGATGAACACGCA*GTCTTC*CACGGGTAGCGATTTCAGGA G-3′ (*BamH*I site, His tag, and *Bbs*I site are indicated as bold, underlined, and italic, respectively). The PCR fragment containing Lpp-OmpA gene was ligated into pGEM-T vector to produce pLpp-OmpA. Into the pLpp-OmpA digested with *Bbs*I, the *Bbs*I-*Fok*I fragment carrying the multimeric Buf IIIb-L gene isolated from pMBT-B_n_ was cloned, producing pLpp-OmpA-B_n_ (n = number of a Buf IIIb-L, 0, 1, 2, and 3). The number of monomers in each vector was confirmed by cleaving the vector with *Not*I, whose sites flank each multimer. The DNA fragment of Lpp-OmpA-multimeric Buf IIIb-L was isolated after digestion with *Nde*I and *BamH*I from pLpp-OmpA-B_n_, and ligated into the expression vector pET21c digested with the same enzymes, generating pLO-B_n_ (Fig. 1).

### Expression of Lpp-OmpA-multimeric Buf IIIb-L fusion genes in *E. coli*

*E. coli* BL21 (DE3) cells were transformed with expression vectors containing the Lpp-OmpA-multimeric Buf IIIb-L fusion genes. Each transformant harboring pLO-B_n_ (n = 0, 1, 2, and 3) was inoculated into 3 ml of LB supplemented with ampicillin (50 µg/ml), and grown at 37°C for 9 to 12 h. Each culture was then diluted (1∶100) into fresh medium and grown at 37°C. At an OD_600_ = 0.6, isopropyl-β-D-thio-galactoside (IPTG) was added to a final concentration of 0.2 mM for the induction of fusion genes. The cells were harvested 4 h after induction by centrifugation at 6,000 × g for 10 min at 4°C, and lysed by sonication (6×30 s, B. Braun instruments, Allentown, PA, USA) on ice. The amount of LO-B_n_ fusion proteins in the whole cell lysates was determined by quantifying the protein bands in each lane of 10% sodium dodecyl sulfate (SDS)-polyacrylamide gels by densitometry at 600 nm (Bio/Profile image analysis software; Bio-1D, Vilber Lourmat, France). The presence of LO-B_n_ fusion proteins in the whole cell lysates was also confirmed by Western blot using anti-His antibody (Santa Cruz Biotechnology, Santa Cruz, CA, USA).

### Immunofluorescence microscopy

One ml of *E. coli* BL21 (DE3) cells harboring pLO-B_n_ (n = 0, 1, 2, and 3) was harvested by centrifugation at 6,000× g for 10 min at 4°C, washed with 10 mM NAPB, and resuspended in the same buffer. Cells were then incubated with anti-His-FITC antibody (Santa Cruz Biotechnology) diluted (1∶200) in NAPB for 1 h at room temperature. Prior to microscopic observation, cells were washed five times with 10 mM NAPB to remove unbound anti-His-FITC antibody, then mounted on microscopic slides and examined by confocal microscopy. Confocal images were acquired with a confocal scanning laser Zeiss LSM 510 microscope (Jena, Germany) equipped with a 100× objective. The z-stack image of *E. coli* cells harboring pLO-B_3_ was collected as 0.2 micron z-stacks using the same confocal microscope with a 400× objective. Fluorophores were excited with an argon laser (488 nm) for FITC.

### Fractionation of outer membrane proteins

One hundred ml of each culture broth was centrifuged at 6,000× g for 10 min at 4°C, and the cell pellets were washed with 25 mM Tris-HCl buffer (pH 8.0), followed by centrifugation at 6,000× g for 10 min at 4°C. The cell pellets (1×10^11^ each recombinant cells) were then resuspended in 25 mM Tris-HCl buffer (pH 8.0) containing 50 µg/ml of lysozyme, incubated for 1 h on ice; cells were and disrupted by sonication. The lysates were centrifuged at 10,000× g for 15 min at 4°C to remove any unbroken cells, and centrifuged again at 115,000× g for 1 h at 4°C to separate the membrane and soluble fraction. The membrane pellets were then resuspended with phosphate-buffered saline (PBS) containing 0.01 mM MgCl_2_ and 2% Triton X-100 for solubilization of the inner membrane. After incubation for 30 min at room temperature, the insoluble pellets containing outer membrane proteins were obtained by centrifugation at 115,000× g for 1 h at 4°C. Outer membrane protein extracts were obtained by washing the insoluble pellets with 25 mM Tris-HCl buffer (pH 8.0), followed by resuspending in 20 µl of the 2% SDS buffer, and analyzed by using 10% SDS-polyacrylamide gel electrophoresis [14], [17], [19], [20]. The LO-B_n_ fusion proteins in the outer membrane protein extracts were quantified by measuring the protein bands of SDS-polyacrylamide gel by densitometry at 600 nm, according to the manufacturer' protocol (Bio/Profile Image Analysis Software; Bio-1D, Vilber Lourmat, France). In brief, the intensity of each protein band was measured as the integrated volume of pixels (with linear dimensions *x* and *y* in millimeters and the *z* axis as the relative absorbance) associated with each Coomassie blue-stained band, and the amount of LO-B_n_ proteins in the outer membrane protein extracts was calculated by multiplying the ratio of the intensity of the LO-B_n_ fusion protein band over the sum of intensities of all protein bands in the same lane of a SDS gel by the total proteins determined above.

### Cleavage of the surface-displayed LO-B_n_ fusion proteins by pepsin

For pepsin digestion, the cell pellets (1×10^11^ each recombinant cells) were resuspended in 1 ml of simulated gastric fluid (SGF; 0.7% (v/v) HCl, 0.2% (v/v) NaCl, and 1000 U of pepsin), and incubated at 37°C for 1 h. The reactions were then stopped by adding 1 ml of 0.7% (v/v) NaOH, 0.2% (v/v) NaCl, and pepsin-digested peptides in the supernatants were recovered by centrifugation at 13,000× g for 15 min at 4°C and analyzed using SDS-polyacrylamide gel electrophoresis. The digested peptides were then applied to a Resource 15S cation exchange column (Pharmacia LKB Biotechnology, Inc., Uppsala, Sweden). The bound peptides were eluted by applying a linear 0 to 1 M NaCl gradient in elution buffer (20 mM 2-[N-morpholino] ethanesulfonic acid, pH 6.0 with 1 M NaCl) and concentrated by lyophilization. The lyophilized peptides were further purified by reversed-phase HPLC on a Delta-Pak C_18_ column (3.9 mm×300 mm, Waters) using a linear elution gradient of 0 to 50% acetonitrile in 0.1% (v/v) trifluoroacetic acid (TFA) at 1 ml/min for 1 h. Synthetic Buf IIIb-L (0-400 µg/ml) was used as control for quantification of the purified recombinant Buf IIIb-L monomer. A calibration curve was constructed by plotting average peak area obtained by HPLC versus concentration of the synthetic peptide. The calibration curve showed excellent linearity (r^2^>0.999) over the concentration range investigated (Y = 4.6457 X+13.174, where Y is the peak-area obtained by HPLC and X is the concentration of the peptide).

### Measurement of susceptibility of oral infectious pathogens to the pepsin-digested peptide mixtures obtained from *E. coli* with surface-expressed LO-B_n_ fusion proteins

Antimicrobial activities of the pepsin-digested peptide mixtures obtained from *E. coli* cells with surface-expressed LO-B_n_ fusion proteins were tested against two oral infectious pathogens, *Salmonella enteritidis* (ATCC 13076) and *Listeria monocytogenes* (ATCC 15313) as described above except using 10 µl of the 20-fold diluted peptide mixtures obtained from 1×10^11^ *E. coli* cells with surface-expressed LO-B_n_ fusion proteins instead of synthetic peptide. Growth of cells was determined by measuring absorbance at 620 nm with a Model 550 Microplate Reader (Bio-Rad). The percentage of viability was determined as follows: Viability (%) = (A_s_−A_0_)/(A_100_−A_0_)×100, where A_s_ is the absorbance of the sample, A_100_ is the absorbance of cells without any treatment, and A_0_ is the absorbance of cells treated with MIC value of synthetic Buf IIIb-L.

## Results

### Buf IIIb derivatives with a pepsin substrate residue at the C-terminus

**Figure 2:**
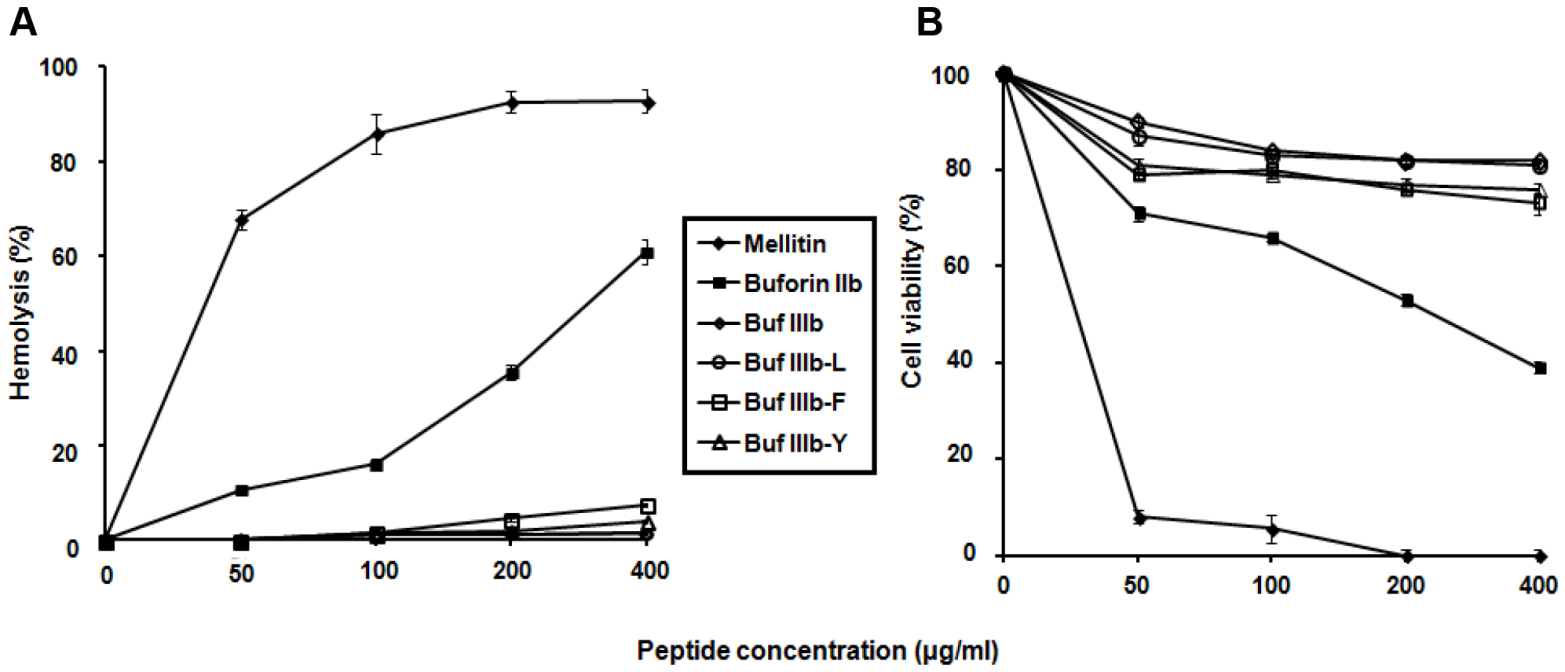
Hemolysis and in vitro cytotoxicity assays. (A) Hemolytic activity. Fresh RBC suspension was incubated with peptides. The release of hemoglobin into the supernatant was monitored to determine membrane damage of RBCs. (B) *In vitro* cytotoxicity. Peptides were added to HaCaT keratinocytes, and cell viability was measured by the MTT assay after a 24-h incubation with the peptides. Data in (A) and (B) represent Data in (A) and (B) represent the mean±SD of 3 independent experiments.

Earlier experiments disclosed that Buf IIIb exhibits potent antimicrobial activity via targeting intracellular components, such as DNA in microorganisms, and does not cause damage to mammalian cells at the same time, resulting in a 7-fold improvement in the therapeutic index, compared to its parent antimicrobial peptide, buforin IIb [13]. In this study, we made Buf IIIb derivatives by adding a pepsin substrate residue (L, F, or Y) at the C-terminus of Buf IIIb, thus making them to be released from multimers by pepsin cleavage (Table 1). Significantly, Buf IIIb itself was not cleaved by pepsin, as assessed using PeptideMass software tools on the Expert Protein Analysis System (ExPASy). As shown in Table 2, the addition of pepsin substrate residue (L, F, or Y) at the C-terminus of Buf IIIb led to a ∼2-fold decrease in MIC (0.5–4 µg/ml) compared to the parent peptide Buf IIIb (0.5–2 µg/ml). Among the Buf IIIb derivatives, Buf IIIb-F and Buf IIIb-Y lysed 7.5% and 4.0% of human RBCs (Fig. 2 A) and killed 27% and 24% of HaCaT keratinocytes at 400 µg/ml (Fig. 2 B). On the other hand, Buf IIIb-L, like the parent peptide Buf IIIb, was completely inactive against human RBCs and killed less than 10% of HaCaT keratinocytes at 400 µg/ml. Therefore, Buf IIIb-L was selected for the construction of tandem multimers for *E. coli* cell surface display.

**Table 2:** Antimicrobial activities of Buf IIIb derivatives.

	MIC ( µg/ml)^a^					
Microorganism	Buforin llb	Buf IIIb	Buf IIIb-L	Buf IIIb-F	Buf IIIb-Y	Magainin II
**Gram-positive bacteria**						
*Bacillus subtilis*(ATCC 62037)	1	2	2	2	2	8
*Staphylococcus aureus*(ATCC 15752)	2	2	2	2	2	8
*Streptococcus mutans* (ATCC 25175)	2	2	2	2	2	32
**Gram-negative bacteria**						
*Escherichia coli* (ATCC 27325)	1	1	2	4	4	32
*Pseudomonas putida*(ATCC 17426)	2	1	4	4	2	16
*Salmonella enteritidis*(ATCC 13076)	2	2	4	2	2	16
**Fungi**						
*Candida albicans*(ATCC 10231)	2	1	2	0.5	4	4
*Saccharomyces cerevisiae*(ATCC 44774)	2	0.5	2	0.5	1	4

a The MIC represents the amount of AMP required to inhibit growth of the microorganism. Each MIC was determined from two independent experiments performed in triplicates.

### Construction and expression of Lpp-OmpA-multimeric Buf IIIb-L fusion genes

**Figure 3:**
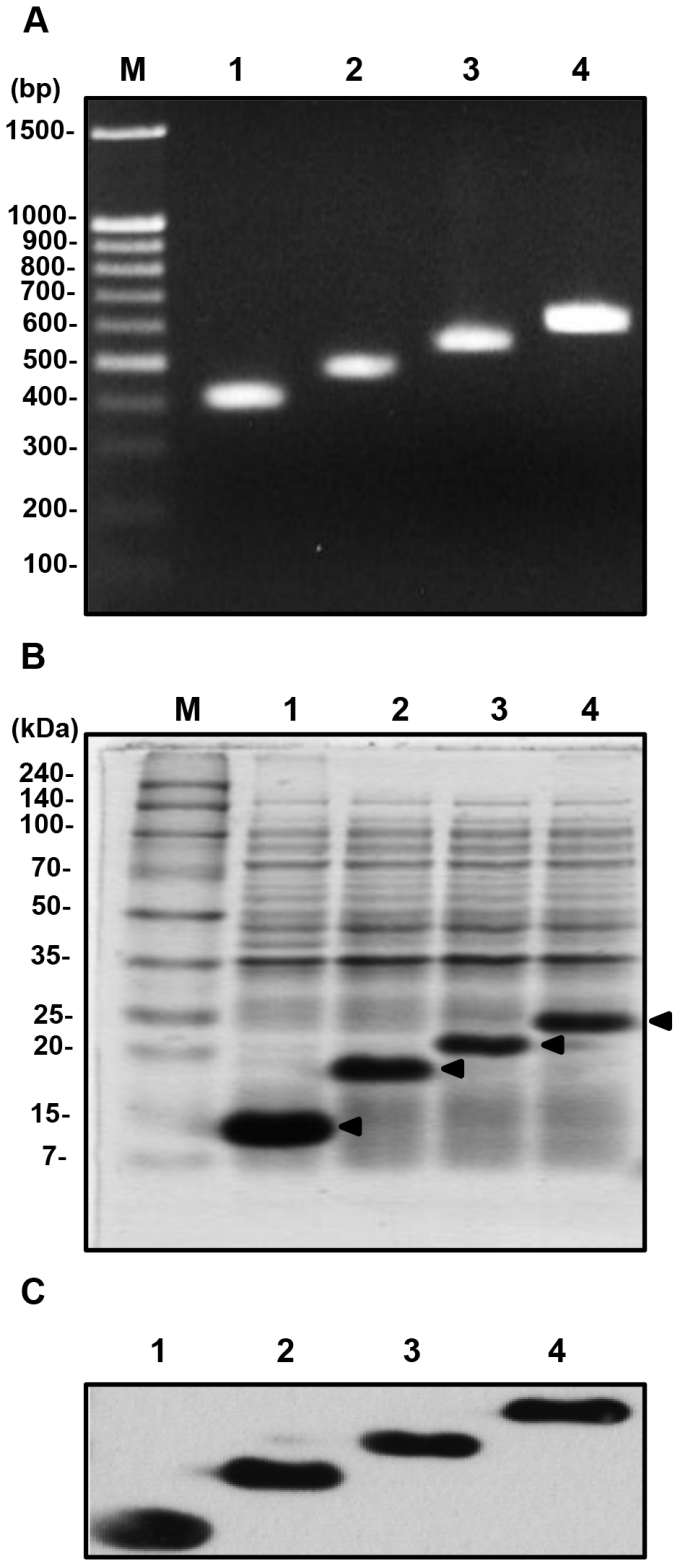
Confirmation of Lpp-OmpA-multimeric Buf IIIb-L fusion genes and the expression of LO-B_n_ fusion proteins. (A) The number of Buf IIIb-L genes cloned in pLpp-OmpA-B_n_ was determined by digesting with *Not*I, whose sites flank each multimer. Lanes 1–4 represent *Not*I-digested pLpp-OmpA-B_0_, -B_1_, -B_2_, and -B_3_, which contains 0, 1, 2, or 3 copies of the Buf IIIb-L gene, respectively. Lane M represents size markers. (B) Total cell proteins were analyzed using SDS-PAGE. Lanes 1–4 represent total cell proteins from *E. coli* BL21 (DE3) harboring pLO-B_0_, -B_1_, -B_2_, and -B_3_, respectively. Lane M represents molecular weight markers. The *closed triangles* indicate the LO-B_n_ fusion proteins expressed from each clone. (C) The presence of LO-B_n_ fusion proteins in the total cell proteins from each clone was confirmed by Western blot using anti-His antibody. The bands corresponding to each LO-B_n_ fusion proteins (n = 0, 1, 2, and 3) were detected in lanes 1–4 (13.1, 15.85, 18.60, and 21.35 kDa, respectively).

To express Buf IIIb-L as tandem multimers on the surface of *E. coli*, Buf IIIb-L gene was multimerized and subsequently fused with the gene encoding an anchor protein, Lpp-OmpA (Fig. 1). The multimerization of Buf IIIb-L gene was performed as described by Kim *et al.* using two class IIS enzymes, *Bbs*I and *Fok*I [21]. The clones, pMBT-B_1_, -B_2_, and B_3_, each containing 1, 2, and 3 copies of Buf IIIb-L gene, respectively, were selected. The *Bbs*I and *Fok*I fragment of these clones were then cloned into pLpp-OmpA that has an chimeric Lpp-OmpA gene consisting of the signal sequence and the first nine amino acids of lipoprotein (Lpp), residues 46-159 of outer membrane protein OmpA, and His tag (histidine 6-mer), producing pLpp-OmpA-B_n_ (n = number of a Buf IIIb-L, 0, 1, 2, and 3). The number of Buf IIIb-L genes cloned in pLpp-OmpA-B_n_ was determined by digesting with *Not*I, whose sites flank each multimer (Fig. 3A). The DNA fragment of Lpp-OmpA-multimeric Buf IIIb-L was then isolated after digestion with *Nde*I and *BamH*I from pLpp-OmpA-B_n_, and ligated into the expression vector pET21c digested with the same enzymes, generating pLO-B_n_. The successful expression of LO-B_n_ fusion proteins were verified by SDS-polyacrylamide gel electrophoresis and Western blot analysis (Fig. 3B, C).

### Confirmation of LO-B_n_ fusion proteins displayed on the cell surface

**Figure 4:**
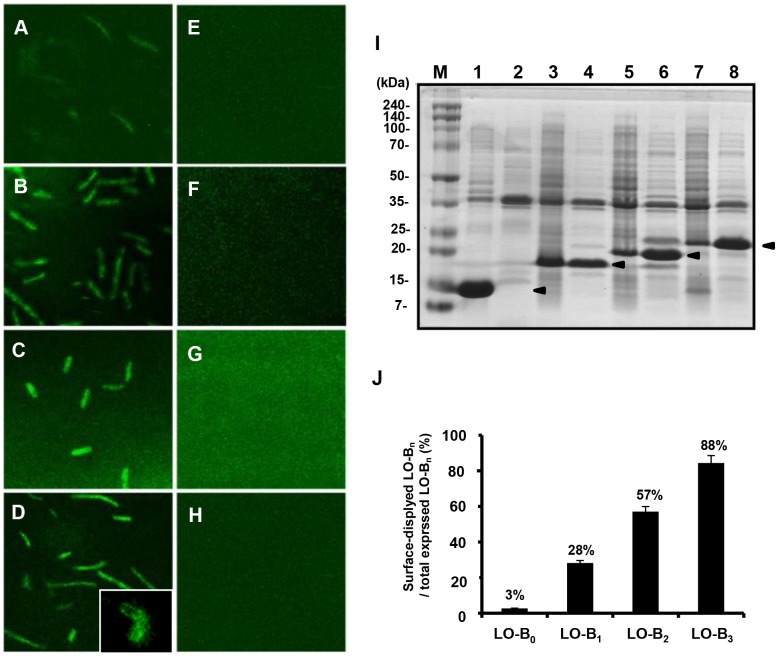
Confirmation of LO-B_n_ fusion proteins displayed on the cell surface. Cells harboring pLO-B_0_, -B_1_, -B_2_, and -B_3_ were incubated with anti-His-FITC antibody diluted (1∶200) in NAPB for 1 h at room temperature. Confocal images show the localization of LO-B_0_ (Lpp-OmpA, A), LO-B_1_ (B), LO-B_2_ (C), and LO-B_3_ fusion proteins (D), respectively. No fluorescence signal was detected when the same cells were not induced by IPTG (E–H). The z-stack confocal image (D, inset) clearly shows the surface location of LO-B_3_ fusion proteins as a concentrated fluorescent ring along the cell surface. (I) SDS-PAGE analysis of the outer membrane proteins and inclusion bodies. Lanes 1, 3, 5, and 7 represent inclusion bodies in the cytoplasm of *E. coli* BL21 (DE3) harboring pLO-B_0_, -B_1_, -B_2_, and -B_3_, respectively. Lanes 2, 4, 6, and 8 represent the outer membrane proteins isolated from *E. coli* BL21 (DE3) harboring pLO-B_0_, -B_1_, -B_2_, and -B_3_, respectively. Lane M represents molecular weight markers. The *closed triangles* indicate the LO-B_n_ fusion proteins. (J) The ratio of surface-displayed LO-B_n_ fusion proteins to total expressed LO-B_n_ fusion proteins.

The goal of our study was to express LO-B_n_ fusion proteins on the surface of *E. coli* to make it as an antimicrobial microorganism. Therefore, we first determined whether LO-B_n_ fusion proteins were successfully displayed on the surface of *E. coli*. To determine the precise localization of LO-B_n_ fusion proteins, immunofluorescence labeling of cells was performed by incubation with anti-His-FITC antibody. As shown in Fig. 4, *E. coli* cells harboring pLO-B_1_, -LO-B_2_, or -LO-B_3_ were observed as solid fluorescent rods when induced with IPTG, indicating the presence of LO-B_n_ fusion proteins on the surface (Fig. 4B–D). Fluorescence of cells harboring pLO-B_0_, which express only Lpp-OmpA anchor proteins, was relatively weak compared to the others (Fig. 4A). On the other hand, no fluorescence signal was detected when the same cells were not induced by IPTG (Fig. 4E–H).

**Figure 5:**
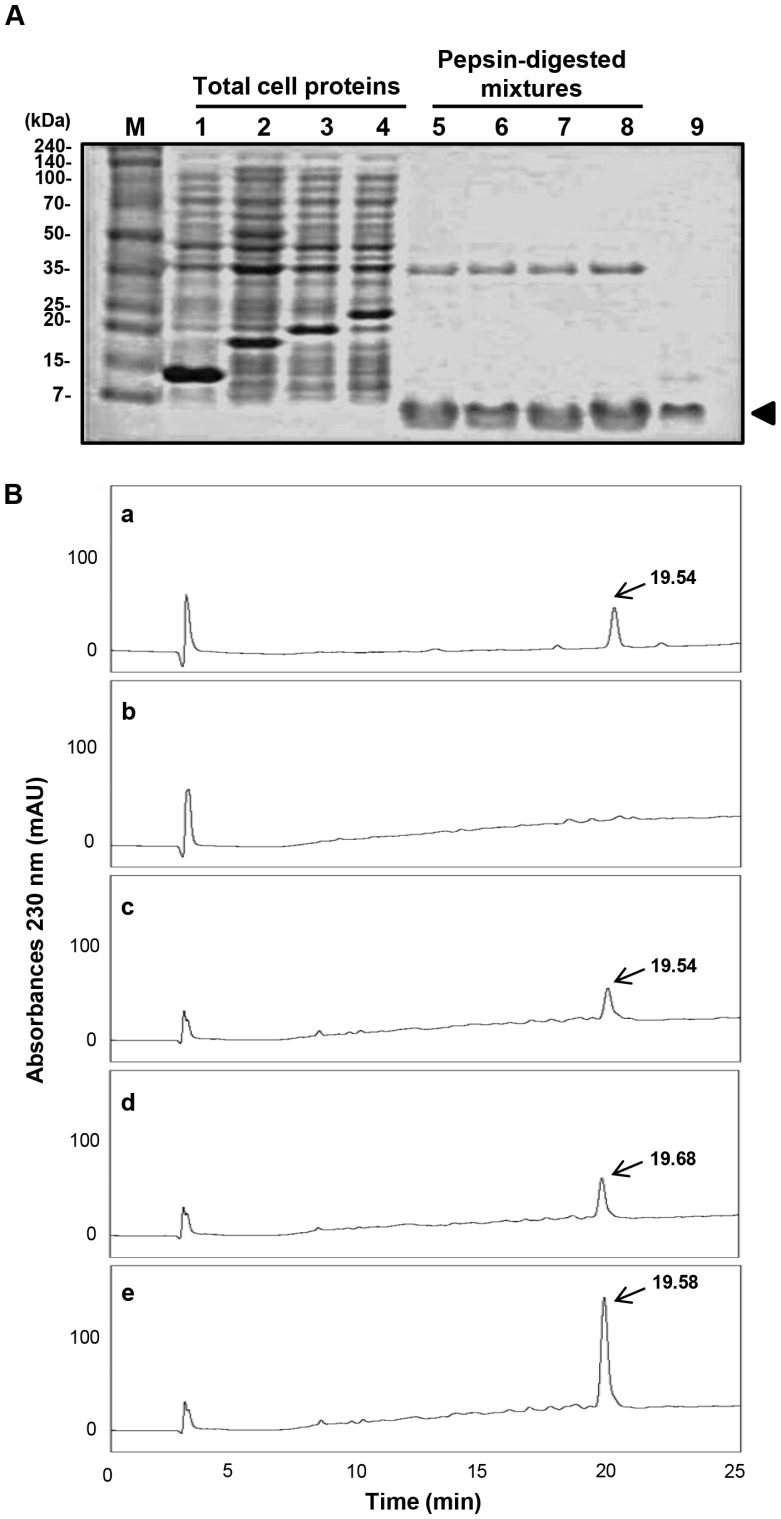
Pepsin cleavage of the surface-displayed LO-B_n_ fusion proteins. (A) SDS-PAGE analysis of the pepsin-digested mixtures. Lanes 1–4 represent total cell proteins, and lanes 5–8 represent the pepsin-digested mixtures from *E. coli* BL21 (DE3) harboring pLO-B_0_, -B_1_, -B_2_, and -B_3_, respectively. Lane 9 represents the synthetic Buf IIIb-L, and lane M represents molecular weight markers. The *closed triangle* indicates a band corresponding to Buf IIIb-L monomer. (B) HPLC-chromatograms of the synthetic Buf IIIb-L (a) and the purified recombinant Buf IIIb-L monomers from the pepsin-digested mixtures from *E. coli* BL21 (DE3) harboring pLO-B_0_, -B_1_, -B_2_, and -B_3_, respectively (b–d). The *arrow* indicates a peak corresponding to Buf IIIb-L monomer.

We also fractionized the outer membrane proteins and inclusion bodies from the cells harboring pLO-B_n_ (n = 0, 1, 2, and 3) and analyzed them by SDS-polyacrylamide gel electrophoresis (Fig. 5I). The bands corresponding to each LO-B_n_ fusion protein (13.1, 15.85, 18.60, and 21.35 kDa, respectively) were detected in the outer membrane protein extracts and inclusion body extracts obtained from the cells harboring pLO-B_n_. The ratio of surface-displayed LO-B_n_ fusion proteins to the total expressed LO-B_n_ fusion proteins increased as the number of Buf IIIb-L monomer attached to Lpp-OmpA anchor protein increased. 88% of the total expressed LO-B_3_ fusion proteins were displayed on the cell surface, while the percentage of successfully displayed LO-B_2_, LO-B_1_, and Lpp-OmpA were 57%, 28%, and 3%, respectively (Table 3 and Fig. 4J).

**Table 3:** The amounts of the LO-B_n_ fusion proteins and the HPLC-purified recombinant Buf IIIb-L monomers obtained from 1×10^11^ cells of *E. coli*.

Strain	Total proteins ( µg)^a^	Total expressedLO-B_n_ ( µg)^b^	Surface-displayedLO-B_n_ ( µg)^b^	Buf IIIb-L ( µg)^c^
*E. coli* harboring pLO-B_1_	37800	5818	1629	85.58
*E. coli* harboring pLO-B_2_	38065	4058	2313	94.42
*E. coli* harboring pLO-B_3_	32886	3631	3195	251.93

a Total protein concentration was determined by BCA protein assay using bovine serum albumin as a standard.

b The amounts of the total expressed LO-B_n_ fusion proteins and the surface displayed LO-B_n_ fusion proteins were determined by quantifying the protein bands of SDS-polyacrylamide gels by densitometry at 600 nm as described in Materials and Methods.

c The amounts of the HPLC-purified recombinant Buf IIIb-L monomers were calculated using the calibration curve constructed by plotting the average peak area obtained by HPLC versus the concentration of the synthetic Buf IIIb-L peptide.

### Cleavage of the surface-displayed LO-B_n_ fusion proteins by pepsin

To gain the antimicrobial activity, free Buf IIIb-L monomers should be released from the surface-displayed LO-B_n_ fusion proteins by pepsin-mediated cleavage. To test if pepsin can cleave the fusion protein *in vitro*, the cell pellets (1×10^11^ each recombinant cells) were resuspended in simulated gastric fluid, and incubated at 37°C for 1 h. As shown in Fig. 5A, pepsin cleaved the fusion proteins and generated a band corresponding to Buf IIIb-L monomers. The digested peptides were further purified by cationic exchange chromatography and RP-HPLC. The HPLC chromatograms of the recombinant Buf IIIb-L monomers purified from the pepsin-digested peptide mixture showed the same retention time with that of synthetic Buf IIIb-L (Fig. 5B). The amounts of the purified recombinant Buf IIIb-L monomer obtained from *E. coli* with surface-expressed LO-B_n_ fusion proteins were calculated using the calibration curve constructed by plotting the average peak area obtained by HPLC versus the concentration of the synthetic Buf IIIb-L peptide. We got 85.58 µg, 94.42 µg, and 251.93 µg of recombinant Buf IIIb-L monomers from the 1×10^11^ *E. coli* cells with surface-expressed LO-B_1_, -B_2_, and -B_3_ fusion proteins, respectively (Table 3). The recombinant Buf IIIb-L monomers exhibited identical antimicrobial activity to chemically synthesized Buf IIIb-L against eight representative microorganisms (data not shown) and two oral infectious pathogens of livestock *S. enteritidis* (4 µg/ml of MIC) and *L. monocytogenes* (2 µg/ml of MIC).

### Susceptibility of oral infectious pathogens to the pepsin-digested peptide mixtures obtained from *E. coli* with surface-expressed LO-B_n_ fusion proteins

**Figure 6:**
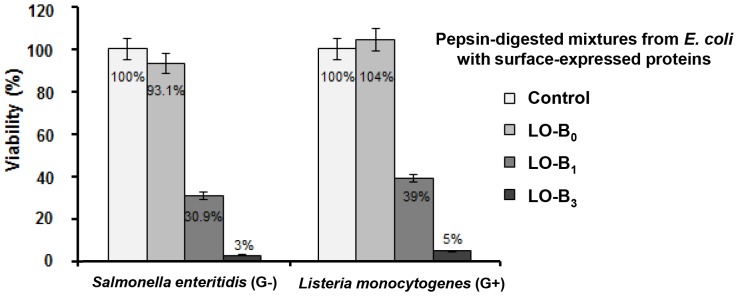
Susceptibility of oral infectious pathogens to the pepsin-digested peptide mixtures obtained from *E.* *coli with surface-expressed LO-B_1_ and -B_3_ fusion proteins.* Antimicrobial activities of the pepsin-digested peptide mixtures from *E. coli* with surface-expressed LO-B_1_ and -B_3_ fusion proteins were investigated against *S. enteritidis* and *L. monocytogenes*. The pepsin-digested mixtures obtained from *E. coli* without surface-expressed protein were used as a negative control. Growth inhibition of pathogens was determined by measuring the absorbance at 620 nm.

To verify the effectiveness of using *E. coli* with surface-expressed LO-B_n_ fusion proteins as antimicrobial microorganism in the stomach of livestock against oral infectious pathogens, we measured the susceptibility of oral infectious pathogens to the pepsin-digested peptide mixtures obtained from 1×10^11^ *E. coli* cells with surface-expressed LO-B_1_ and -B_3_ fusion proteins. As shown in Fig. 6, the viabilities of *S. enteritidis* and *L. monocytogenes* were significantly reduced by the pepsin-digested peptide mixtures (diluted 20-fold) obtained from *E. coli* with surface-expressed LO-B_1_ fusion proteins (30.9% and 39%, respectively). The pepsin-digested mixture obtained from *E. coli* with surface-expressed LO-B_3_, which contained approximately 3-fold amounts of recombinant monomeric Buf IIIb-L than LO-B_1_ (Table 3), almost completely inhibited the growth of both bacteria. On the other hand, the pepsin-digested mixtures obtained from *E. coli* without surface-expressed proteins or *E. coli* with surface-expressed Lpp-OmpA did not show any inhibitory effect.

## Discussion

Antibiotics have been used as therapeutics and prophylactic treatment to control a variety of bacterial infection clearance in the livestock production for more than 60 years. Many types of antibiotics have also been fed at non-therapeutic dosage in the livestock production to increase productivity and feed consumption of animals. However, the use of antibiotics in the livestock production, 100 to 1000 times in terms of annual quantities that in the human population, has been suspected as a major contributor to the emergence of antibiotic-resistant bacteria [22]. Thus many countries have banned the administration of conventional antibiotics (at non-therapeutic doses), as feed additives to livestock [6], [7]. This situation has spurred herculean efforts to develop alternative antibiotics for livestock. AMPs have been regarded as a potential solution to the worldwide emergence and rapid horizontal spread of antibiotic-resistant traits in bacteria of human and veterinary clinical significance [23]. The livestock industry, however, has not shown a great deal of interest in developing alternative antibiotics base on AMPs. The main reason is undoubtedly the high cost of manufacturing peptides on a large scale and in a pure form to be used for livestock.

Here we developed the most cost effective means of providing AMPs to livestock by consisting rapid-acting and potent antimicrobial *E. coli* displaying multimeric Buf IIIb-L, a potent AMP without cytotoxicity, on the cell surface, and showed the effectiveness of using this antimicrobial *E. coli* as a whole cell antibiotics in the stomach of livestock against oral infectious pathogens. To make a live antimicrobial microorganism, a two-step strategy consisting cell-surface display and enzyme-controlled activation was explored; (1) Buf IIIb is expressed as inactive Lpp-OmpA-fused tandem multimers on the *E. coli* cell surface with a pepsin substrate residue L at the C-terminus of each monomer, (2) the fusion protein is cleaved by pepsin in the stomach of livestock, restoring the antimicrobial activity upon the release of free Buf IIIb-L monomers. We constructed the fusion proteins to release Buf IIIb-L monomers in the stomach of livestock, thus they can act against foodborne pathogens infected via the oral route. The acidic conditions in the stomach are generally considered a low risk for transmission of pathogenic bacteria, but the foodborne pathogens such as *S. enteritidis* and *L. monocytogenes* can survive at pH as low as 2.0 and persist for up to several weeks when inoculated in acidic environment [24]. After passing through the stomach and reaching the small intestine, these bacteria penetrate the intestinal mucosa and accumulate in the lymph nodes, where they multiply and disseminate to the spleen and liver [25], [26]. Therefore, killing foodborne pathogens in the stomach before reaching the small intestine is significant.

As expected, the Lpp-OmpA-multimeric Buf IIIb-L (LO-B_n_) fusion proteins did not affect the growth of host *E. coli*, and successfully displayed on the cell surface (Fig. 4). Upon cleavage by pepsin, the fusion proteins displayed on the surface of *E. coli* were converted into active AMP monomers, and the liberated Buf IIIb-L monomers inhibited the growth of *S. enteritidis* and *L. monocytogenes* (Figs. 5 and 6). These data indicate that the *E. coli* with surface-expressed LO-B_n_ fusion proteins may be used as whole-cell antibiotics in the stomach of livestock against oral infectious pathogens. Importantly, the liberated Buf IIIb-L may act only in the stomach and not affect the normal flora in the intestine, because the peptides will be degraded by trypsin or chymotrypsin there. In fact, Buf IIIb-L was almost completely digested by 30-min incubation with trypsin and chymotrypsin *in vitro*, and lost antimicrobial activities (Fig. S1 and Table S1 in File S1). We are now evaluating the effectiveness of *E. coli* with surface-expressed LO-B_n_ as whole-cell antibiotics *in vivo* using mouse model infected orally with either 1×10^8^ CFU of *L. monocytogenes* or *S. enteritidis* [27]. Preliminary results showed that mice treated with 1×10^11^ *E. coli* with surface-expressed LO-B_3_, when administrated orally 2 h after pathogen infection, survived longer than PBS-treated control mice (data not shown). This increase in survival rate was not seen with the same number of *E. coli* with surface-expressed LO-B_1_, probably because there was not enough amount of recombinant monomeric Buf IIIb-L to be effective against pathogens in the 1×10^11^ cells. In fact, LO-B_3_ produces approximately 3-fold amounts of recombinant monomeric Buf IIIb-L than LO-B_1_. The amounts of the HPLC purified recombinant Buf IIIb-L obtained from 1×10^11^ *E. coli* with surface-expressed LO-B_3_ and LO-B_1_ were 251.93 µg and 85.58 µg, respectively (Table 3). In other words, we could use smaller number of *E. coli* with surface-expressed LO-B_3_ than *E*. *coli* with surface-expressed LO-B_1_ to get the same effect, which is important, because *E. coli* in itself might cause infectious disease. Usually, *E. coli* forms a beneficial symbiotic relationship with its host and plays important roles in promoting the stability of the luminal microbial flora and in maintaining normal intestinal homeostasis [28]. However, immune-suppressed host, or when the gastrointestinal barriers are damaged, some *E. coli* can cause infectious disease [29]. Therefore, ongoing studies are aimed to surface-display tandem multimeric AMPs on GRAS (Generally Recognized As Safe) strains such as *Lactobacillus* [30].

Overall, our novel strategy using microorganism with AMPs displayed on the cell surface as whole-cell antibiotics may represent the most effective means of providing potent AMPs to livestock, and have a great impact on controlling over pathogenic microorganisms in the livestock production.

## Supporting Information

File S1 **Figure S1. Protease digestion of synthetic BufIIIb-L. 2 µg of Buf IIIb-L was incubated with pepsin (0.4 µg), trypsin (0.1 µg), or chymotrypsin (0.2 µg), respectively, in the digestion buffer recommended by the supplier at 37°C.** At the designated time points, the digestion mixture was sampled and analyzed by 16.5% tricine SDS-PAGE. Table S1. Antimicrobial activities of enzyme-digested Buf IIIb-L.
(ZIP)
