# Supplementary material for: Display of Multimeric Antimicrobial Peptides on the Escherichia coli Cell Surface and Its Application as Whole-Cell Antibiotics
Source: PLoS One. 2013 Mar 14;8(3):e58997. doi: 10.1371/journal.pone.0058997 (PMC3597565; doi:10.1371/journal.pone.0058997)
Supplement: File S1 — Figure S1. Protease digestion of synthetic BufIIIb-L. 2 µg of Buf IIIb-L was incubated with pepsin (0.4 µg), trypsin (0.1 µg), or chymotrypsin (0.2 µg), respectively, in the digestion buffer recommended by the supplier at 37°C. At the designated time points, the digestion mixture was sampled and analyzed by 16.5% tricine SDS-PAGE. Table S1. Antimicrobial activities of enzyme-digested Buf IIIb-L. (ZIP) [file pone.0058997.s001.zip › Table S1.docx]

**Table S1. Antimicrobial activities of enzyme-digested Buf IIIb-L**

|  | MIC (μg/ml)^a^ | | | |
| --- | --- | --- | --- | --- |
| Microorganism | Buf IIIb-L digested with | | |  |
|  | Pepsin | Trypsin | Chymotrypsin |  |
| **Gram-positive bacteria** |  |  |  |  |
| *Bacillus subtilis*  (ATCC 62037) | 2 | >256 | >256 |  |
| *Staphylococcus aureus*  ( ATCC 15752) | 2 | >256 | >256 |  |
| *Streptococcus mutans*  (ATCC 25175) | 2 | >256 | >256 |  |
| **Gram-negative bacteria** |  |  |  |  |
| *Escherichia coli*  (ATCC 27325) | 2 | >256 | >256 |  |
| *Pseudomonas putida*  (ATCC 17426) | 4 | >256 | >256 |  |
| *Salmonella enteritidis*  (ATCC 13076) | 4 | >256 | >256 |  |
| **Fungi** |  |  |  |  |
| *Candida albicans*  (ATCC 10231) | 2 | >256 | >256 |  |
| *Saccharomyces cerevisiae*  (ATCC 44774) | 2 | >256 | >256 |  |

^a^ To determine the effect of protease digestion on the antimicrobial activity of Buf IIIb-L, synthetic Buf IIIb-L was first incubated with pepsin, trypsin, or chymotrypsin. After incubation at 37 °C for 30 min, the antimicrobial activity of the digested peptide was determined against eight representative microorganisms, including Gram-positive and Gram-negative bacteria and fungi, as described in Materials and Methods.
