# Supplementary figures and images for: Display of Multimeric Antimicrobial Peptides on the Escherichia coli Cell Surface and Its Application as Whole-Cell Antibiotics
Source: PLoS One. 2013 Mar 14;8(3):e58997. doi: 10.1371/journal.pone.0058997 (PMC3597565; doi:10.1371/journal.pone.0058997)

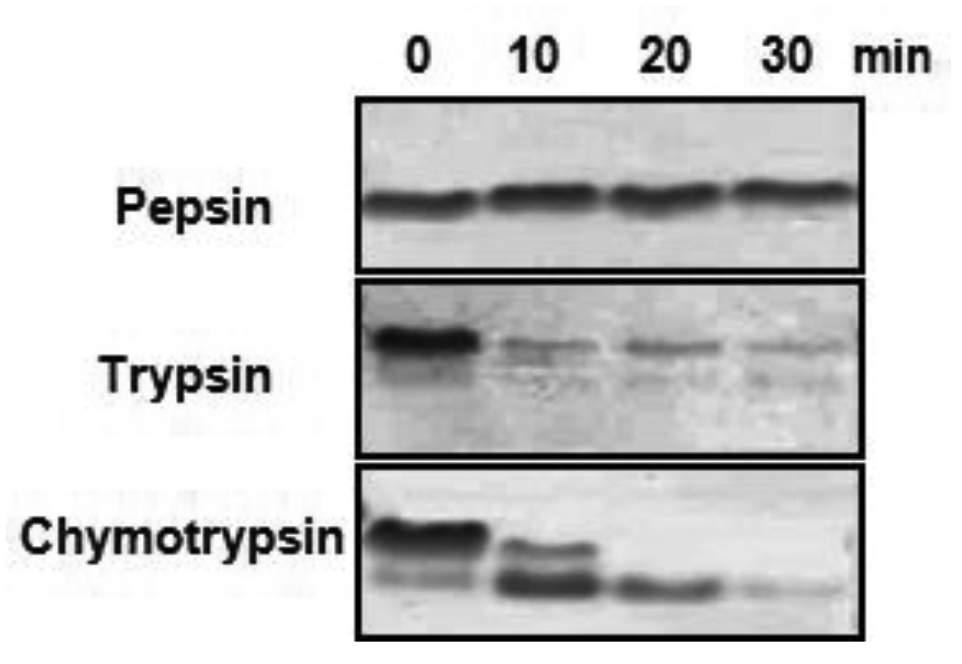

Supplement: File S1 — Figure S1. Protease digestion of synthetic BufIIIb-L. 2 µg of Buf IIIb-L was incubated with pepsin (0.4 µg), trypsin (0.1 µg), or chymotrypsin (0.2 µg), respectively, in the digestion buffer recommended by the supplier at 37°C. At the designated time points, the digestion mixture was sampled and analyzed by 16.5% tricine SDS-PAGE. Table S1. Antimicrobial activities of enzyme-digested Buf IIIb-L. (ZIP) [file pone.0058997.s001.zip › Figure S1.tif]
